# Large Circular Plasmids from Groundwater Plasmidomes Span Multiple Incompatibility Groups and Are Enriched in Multimetal Resistance Genes

**DOI:** 10.1128/mBio.02899-18

**Published:** 2019-02-26

**Authors:** Ankita Kothari, Yu-Wei Wu, John-Marc Chandonia, Marimikel Charrier, Lara Rajeev, Andrea M. Rocha, Dominique C. Joyner, Terry C. Hazen, Steven W. Singer, Aindrila Mukhopadhyay

**Affiliations:** a Biological Systems and Engineering, Lawrence Berkeley National Laboratory, Berkeley, California, USA; b Graduate Institute of Biomedical Informatics, College of Medical Science and Technology, Taipei Medical University, Taipei, Taiwan; c Environmental Genomics and Systems Biology Division, Lawrence Berkeley National Laboratory, Berkeley, California, USA; d Molecular Biophysics and Integrated Bioimaging Division, Lawrence Berkeley National Laboratory, Berkeley, California, USA; e Biosciences Division, Oak Ridge National Laboratory, Oak Ridge, Tennessee, USA; f Department of Civil and Environmental Engineering, University of Tennessee, Knoxville, Tennessee, USA; Cornell University; University of Tennessee at Knoxville; Washington University

**Keywords:** antibiotic resistance, *mer*, mercury resistance, metal resistance, native plasmids, plasmidome

## Abstract

Naturally occurring plasmids constitute a major category of mobile genetic elements responsible for harboring and transferring genes important in survival and fitness. A targeted evaluation of plasmidomes can reveal unique adaptations required by microbial communities. We developed a model system to optimize plasmid DNA isolation procedures targeted to groundwater samples which are typically characterized by low cell density (and likely variations in the plasmid size and copy numbers). The optimized method resulted in successful identification of several hundred circular plasmids, including some large plasmids (11 plasmids more than 50 kb in size, with the largest being 1.7 Mb in size). Several interesting observations were made from the analysis of plasmid DNA isolated in this study. The plasmid pool (plasmidome) was more conserved than the corresponding microbiome distribution (16S rRNA based). The circular plasmids were diverse as represented by the presence of seven plasmid incompatibility groups. The genes carried on these groundwater plasmids were highly enriched in metal resistance. Results from this study confirmed that traits such as metal, antibiotic, and phage resistance along with toxin-antitoxin systems are encoded on abundant circular plasmids, all of which could confer novel and advantageous traits to their hosts. This study confirms the ecological role of the plasmidome in maintaining the latent capacity of a microbiome, enabling rapid adaptation to environmental stresses.

## Abstract (precis)

Plasmidomes have been typically studied in environments abundant in bacteria, and this is the first study to explore plasmids from an environment characterized by low cell density. We specifically target groundwater, a significant source of water for human/agriculture use. We used samples from a well-studied site and identified hundreds of circular plasmids, including one of the largest sizes reported in plasmidome studies. The striking similarity of the plasmid-borne ORFs in terms of taxonomical and functional classifications across several samples suggests a conserved plasmid pool, in contrast to the observed variability in the 16S rRNA-based microbiome distribution. Additionally, the stress response to environmental factors has stronger conservation via plasmid-borne genes as marked by abundance of metal resistance genes. Last, identification of novel and diverse plasmids enriches the existing plasmid database(s) and serves as a paradigm to increase the repertoire of biological parts that are available for modifying novel environmental strains.

## INTRODUCTION

Plasmids are important in horizontal gene transfer and are critical in facilitating genome restructuring by providing a mechanism for distributing genes that provide a selective advantage to their host (1). Typically, plasmids have a modular structure, containing several functional genetic modules. Plasmids are known to vary from 5 to 500 kb in size, although plasmids as small as 2 kb (2–4) to as large as more than 1 Mb in size (5, 6) have been reported. Historically, environmental plasmid studies focused on exogenous isolation of plasmids studied via mating experiments (7, 8) or via plasmid isolation from bacterial strains that can be cultured (9–12). A previous study on groundwater samples revealed the presence of plasmids in strains that could be cultured, indicating the presence of plasmids in bacteria from low-cell-density environments (9). Given that it is well established that only 1% of bacteria on Earth can be readily cultivated (13), a lot remains unexplored, establishing the need to explore plasmids by a cultivation-independent method. More recently, with affordable DNA sequencing technologies, methods have been developed to specifically isolate and sequence circular plasmid DNA. The plasmidome is described to be the entire plasmid content in an environment that is resolved by metagenomic approaches during high-throughput-sequencing experiments (14) and thus circumvents the need to culture environmental bacteria. It identifies all plasmid types—conjugative, mobilizable, and nonmobilizable. Such plasmidome analyses have been performed in cow rumen (15, 16), rat cecum (17), soil (18), and activated sludge (19, 20), samples that are abundant in bacteria. To the best of our knowledge, plasmidomes have not been explored in low-cell-density environments. Due to the role of plasmids in environmental stress adaptation, we explore the plasmids in groundwater typically characterized by low cell counts but diverse and dynamic microbiomes (21). Here we examine samples from the Oak Ridge Field Research Center (ORFRC) site at the Y-12 Federal Security Complex in Oak Ridge, TN, which is a widely studied and characterized model groundwater environment (22–25). To explore the plasmidome of groundwater known to have a fluctuating microbial community along with lowered cell counts (26, 27), we modified known plasmid DNA isolation methods. The goal of this study was to discover the incidence, distribution, and function of plasmids from this site and to develop a foundation to explore the plasmidome of low-cell-density environments. We present the plasmidome analyses from groundwater samples that resulted in the identification of several hundred circular plasmids bearing genes involved in plasmid replication, mobilization, and maintenance along with those that code for metal, antibiotic, and phage resistance, thus bestowing beneficial traits on the host.

## RESULTS AND DISCUSSION

**FIG 1:**
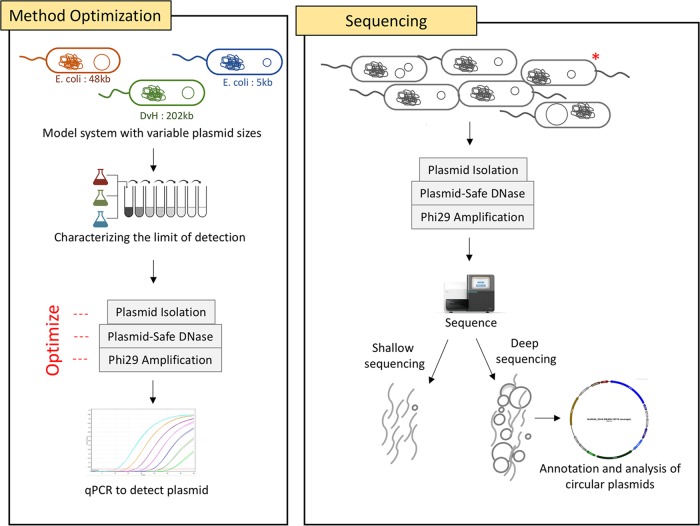
An overview of plasmid DNA isolation and characterization. The plasmid DNA isolation method was optimized using a model system containing two Escherichia coli strains and one Desulfovibrio vulgaris Hildenborough (*Dv*H) with 5-, 48-, and 202-kb plasmid sizes. Successful isolation of plasmid DNA was confirmed by qPCR against a specific plasmid-borne gene. The optimized method was used to isolate plasmid DNA from groundwater samples. The plasmid DNA libraries from certain samples were sequenced individually (deep sequencing), while others were pooled (shallow sequencing). Deep sequencing resulted in identification of several circular plasmids which were further analyzed. *, the model system strains were spiked onto the environmental samples subjected to deep sequencing.

**FIG 2:**
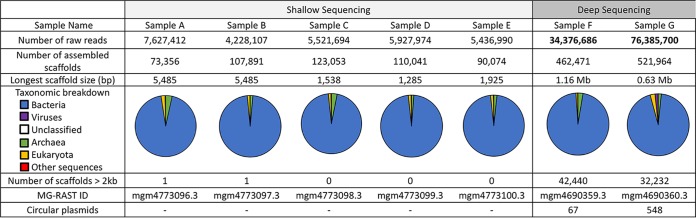
Plasmidome analysis after sequencing of seven groundwater samples from the ORFRC.

The optimized plasmid DNA isolation methodology used in this study yielded the largest plasmid sizes reported in plasmidome studies. Since a previous culture-based study of native plasmids from groundwater environment revealed the presence of a large plasmid (202 kb) (9), we used strains containing large plasmids for the method optimization. The key steps that aided in the optimization of standard protocols (15, 16, 19) were (i) using a model system that contained a large 202-kb plasmid in comparison to the 65-kb (28) and 56-kb (29) plasmids used earlier and (ii) optimization of Phi29 amplification to better represent large plasmids (details in Fig. S1 in the supplemental material). The optimized method was used to isolate plasmid DNA from seven groundwater samples followed by shallow (five plasmid DNA libraries from samples A to E pooled) and deep (two plasmid DNA libraries from samples F and G not pooled) sequencing (Fig. 1). The resulting scaffold distribution showed that the majority of the genes were annotated to be bacterial in origin (Fig. 2). Additionally, the number of raw reads generated was an order of magnitude higher in the deeply sequenced samples, enabling a more comprehensive analysis.

10.1128/mBio.02899-18.1 FIG S1 Optimization of plasmid DNA isolation method. Optimization of plasmid DNA isolation methods performed by plasmid DNA isolation followed by qPCR-ased detection of the plasmid by targeting a unique plasmid-borne gene on each of the plasmids. (a) Threshold values (*C_q_*) obtained when isolating plasmid DNA from the model system containing 5 × 10^5^ cells of each strain using the two alkaline hydrolysis methods, at three different Phi29 incubation temperatures. (b) Optimization of plasmid DNA isolation procedure with bacterial cells present on a filter. A lower *C_q_* number indicates that more copies of the plasmid were present in the DNA sample and hence implies better plasmid detection. The *C_q_* values presented are an average for two technical replicates. Download FIG S1, DOCX file, 0.2 MB. Copyright © 2019 Kothari et al. 2019 Kothari et al. This content is distributed under the terms of the Creative Commons Attribution 4.0 International license.

**FIG 3:**
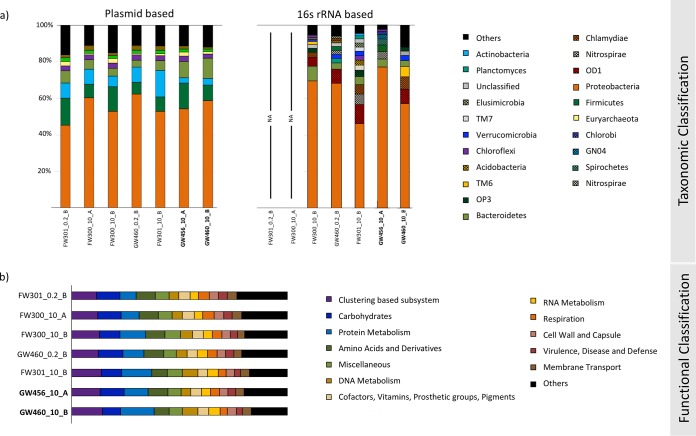
(a) Taxonomic classification (normalized) of “all_ scaffolds” into phyla based on plasmidome (MG-RAST) and 16S rRNA (QIIME) sequences. The MG-RAST analysis was based on the lowest common ancestor. (b) Functional classification (normalized) of “all_ scaffolds” into SEED subsystem categories using MG-RAST (parameters: 1e−5 maximum E value cutoff, 60% maximum identity cutoff, 15-bp minimum alignment length cutoff). It is important to note that the phylogenetic assignment provides the taxonomic classification of the closest known gene homolog and is not indicative of the microbe that originally contained the plasmid at the ORFRC.

We found that the plasmidome sequences across the groundwater samples were more conserved in contrast to the corresponding bacterial taxonomic distribution of these samples (Fig. 3). This pattern might have ecological significance in the role of plasmids in maintaining and transferring conserved key latent functionalities in an ecosystem and has also been reported for plasmidomes from soil and rumen environments (18). The most predominant bacterial phyla represented by ORFs from the groundwater plasmidome were *Bacteroidetes*, *Firmicutes*, *Proteobacteria*, and *Actinobacteria*, with *Proteobacteria* being the most abundantly represented. Interestingly, these phyla are similar to the rumen plasmidome, albeit with a different order of predominance. The most highly represented functional categories (carbohydrates, amino acid metabolism, and clustering-based subsystems) were also similar to that reported in the plasmidome of rumen bacteria (28). This indicates that plasmids from diverse sources dominantly carry genes in similar phylogenetic and functional categories.

Metal and antibiotic resistance genes are one of the most frequently found phenotypic modules carried by bacterial plasmids (30). We found a high abundance of genes annotated to provide resistance to metals—copper, triclosan, arsenic, and mercury—with a large majority being proteobacterial in origin (Table S1). A previous study noted a high abundance of metal resistance genes in these groundwater samples (24) and hypothesized that they might be present on plasmids. The present study confirms that hypothesis. Among antibiotic resistance genes, those providing resistance to aminocoumarin, elfamycin, and bacitracin were predominant (Table S2). Overall, the plasmids were enriched in metal resistance genes compared to the antibiotic resistance genes. There was no detectable metal contamination in our groundwater samples, but given that these groundwater samples are close to a metal-contaminated site at ORFRC (22, 31), it is possible that the microbiome was exposed to metal stress at some point and/or that the dynamic nature of groundwater flow coupled with weather changes might lead to sporadic exposure to low levels of metal contaminants. Accordingly, we find metal resistance genes to be the most predominant, rather than antibiotic resistance genes predominant in activated sludge/wastewater (19, 20), genes required for survival under dairy conditions reported in *Lactococcus* (32), or genes providing an advantage in rumen environments (16).

10.1128/mBio.02899-18.4 TABLE S1 Metal resistance genes abundant in samples F and G. Top hits obtained by the comparison of “all_scaffolds” from samples F and G with the Antibacterial Biocide and Metal Resistance Genes (BacMet) database. The numbers of hits are indicated in parentheses. Abundant metal resistance genes in samples F (a) and G (b) are plotted with the bacterial phyla of the closest reference homolog. Cytoscape was used to generate the figure. Edge thickness indicates the number of genes carried. Colors indicate microbial phyla. Download Table S1, DOCX file, 1.0 MB. Copyright © 2019 Kothari et al. 2019 Kothari et al. This content is distributed under the terms of the Creative Commons Attribution 4.0 International license.

10.1128/mBio.02899-18.5 TABLE S2 Antibiotic resistance encoded by “all_scaffolds” from samples F and G, by comparison with the Antibiotic Resistance Genes Database (ARDB) and the Comprehensive Antibiotic Resistance Database (CARD). Download Table S2, DOCX file, 0.03 MB. Copyright © 2019 Kothari et al. 2019 Kothari et al. This content is distributed under the terms of the Creative Commons Attribution 4.0 International license.

**FIG 4:**
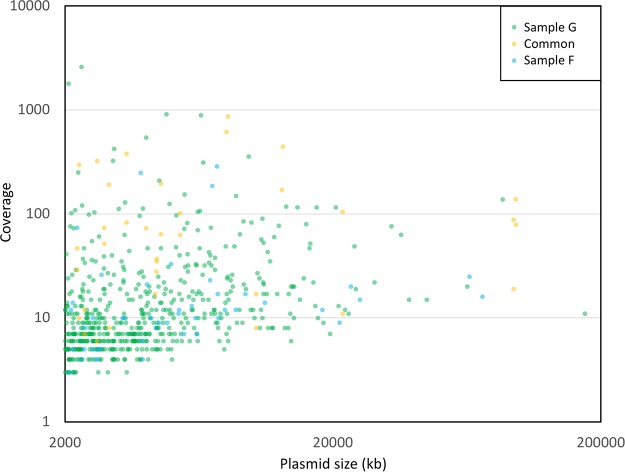
Distribution of circular plasmids from groundwater samples F and G, along with plasmids common to both, based on sequence coverage and plasmid size.

The deeply sequenced groundwater samples resulted in identification of hundreds of complete circular plasmid units of various sizes (67 from sample F and 548 from sample G, Fig. 4). Comparison of the circular plasmids with the ACLAME plasmid database resulted in about 70 to 80% of the ORFs having hits (Table S3), providing further confirmation of the presence of known plasmid-associated genes in our plasmidome data set. Toxin-antitoxin systems important in plasmid maintenance (33, 34) were also reported (Table S4). This study identified several circular plasmids encoding an interesting mix of features such as those providing advantageous traits to the hosts (metal and phage resistance), along with those that help in plasmid maintenance, replication, mobilization, and conjugation (a detailed list of the most abundant plasmids is found in Table S5a and b). Surprisingly, plasmids also carried ORFs annotated to possibly enable phages to invade bacteria (antirestriction protein and a putative phage protein [35]). Additionally, several plasmids were cryptic and could potentially serve as an important source for discovery of novel functional genes and replication systems (17).

10.1128/mBio.02899-18.6 TABLE S3 Top hits obtained by the comparison of “all_scaffolds” and circular plasmids from samples F and G with the A CLAssification of Mobile genetic Elements (ACLAME) database. The number of hits is indicated in the bracket. Download Table S3, DOCX file, 0.01 MB. Copyright © 2019 Kothari et al. 2019 Kothari et al. This content is distributed under the terms of the Creative Commons Attribution 4.0 International license.

10.1128/mBio.02899-18.7 TABLE S4 Percentage of scaffolds carrying homologs of toxin, antitoxin, and regulator gene sequences. Download Table S4, DOCX file, 0.01 MB. Copyright © 2019 Kothari et al. 2019 Kothari et al. This content is distributed under the terms of the Creative Commons Attribution 4.0 International license.

10.1128/mBio.02899-18.8 TABLE S5 (a and b) The top 20 most abundant circular plasmids from samples F (a) and G (b). (c) Eighteen circular plasmids that were shared (>99.8% sequence identity with >93.6% query coverage) between the two samples. Download Table S5, DOCX file, 0.02 MB. Copyright © 2019 Kothari et al. 2019 Kothari et al. This content is distributed under the terms of the Creative Commons Attribution 4.0 International license.

**FIG 5:**
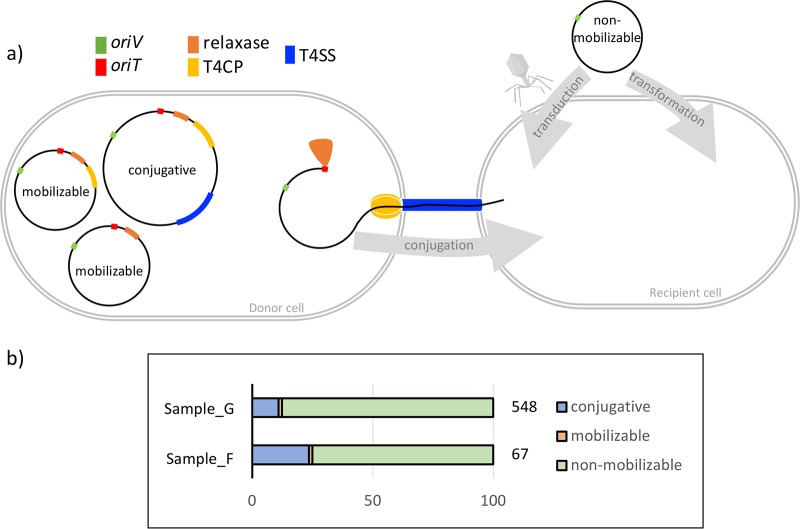
(a) Schematic view of the genetic constitution of plasmids. A nonmobilizable plasmid typically encodes just the origin of vegetative replication (OriV), and the mobilizable plasmid encodes the origin of transfer (OriT) along with relaxase and possibly type IV coupling protein (T4CP). The conjugative plasmid (also called self-transmissible) codes for the type IV secretion system (T4SS) in addition to the above. The nonmobilizable plasmid relies on transformation or transduction for propagation whereas the mobilizable and conjugative plasmids can be propagated via conjugation. The latter process involves relaxase-based cleaving of the plasmid at OriT, followed by interactions with T4CP and T4SS which enable pumping of DNA into the recipient cell. Plasmid types are as described in reference 36. (b) Mobility of circular plasmids from samples F and G depicted as a percentage of the total circular plasmids in that sample.

**FIG 6:**
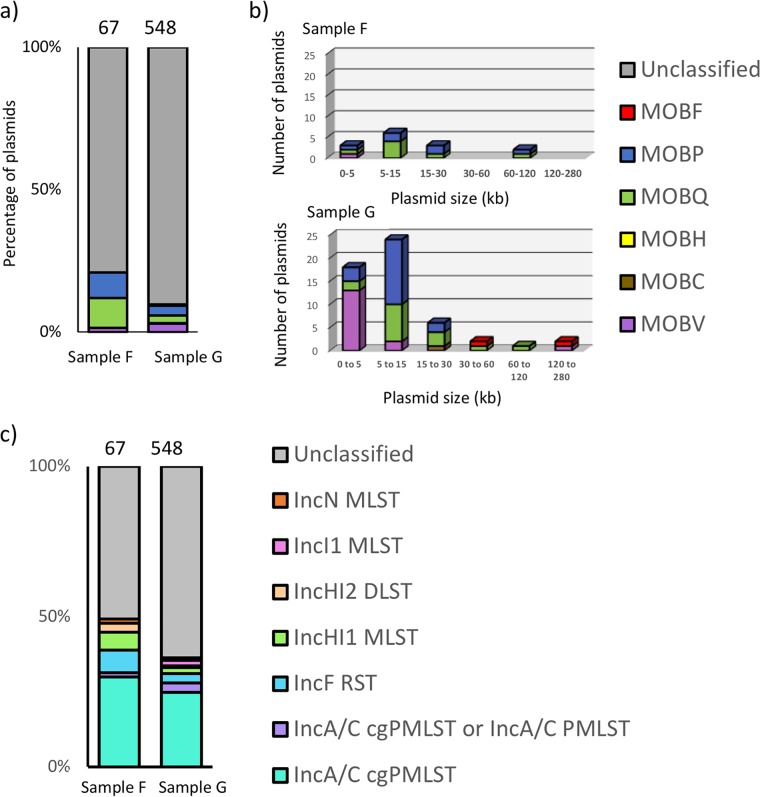
(a and b) Classification of circular plasmids into relaxase/MOB type (a) with the relaxase type plotted as a function of plasmid size (b). (c) Classification of circular plasmids into incompatibility groups.

The circular plasmids from groundwater were diverse in terms of the plasmid type classifications. They were classified as conjugative, mobilizable, or nonmobilizable (Fig. 5). As observed previously (36), the nonmobilizable plasmids were highly predominant in both samples. The circular plasmids from groundwater were diverse in encoding five out of six different relaxase groups and seven incompatibility groups into which plasmids are classified. Based on the relaxase classification (37), MOB_Q_ and MOB_P_ were the most abundant (Fig. 6a). In fact, the relaxase type follows a plasmid size-based distribution (Fig. 6b) as reported earlier. Based on incompatibility classification, plasmids belonging to group IncA/C were highly abundant (Fig. 6c). Interestingly, all the multimetal-resistant plasmids identified in this study were classified as the IncA/C cgPMLST, a group commonly associated with multidrug resistance plasmids (38).

It is noteworthy that this study identified several large plasmids (Fig. 4). The largest circular scaffolds identified were 2.96 and 1.74 Mb from the groundwater samples F and G, respectively. Curiously, the 2.96-Mb scaffold had similarity to known *Ralstonia* and *Pseudomonas* circular phage DNA. Given that this phage DNA was isolated because it was circular, it was removed from further plasmidome analysis but nevertheless emphasizes our methods being optimized for isolation of large circular DNA molecules. The 1.74-Mb plasmid p67 (Table S6) carried several metal resistance genes (for cobalt, zinc, cadmium, and copper) along with genes for plasmid mobilization and conjugation. The plasmid was novel with the closest plasmid reported in literature (Sphingobium baderi DE-13 plasmid pDE1 from an herbicide-manufacturing factory in Kunshan, China [39]) depicting 94% identity but only 10% query coverage. This is one of the highest reported plasmid sizes captured by plasmidome studies (14), further providing evidence that our optimized method was better suited for isolation of large plasmid DNA molecules despite Phi29 amplification biases (29).

10.1128/mBio.02899-18.9 TABLE S6 Homologs of genes carried on the circular plasmid p67. Download Table S6, DOCX file, 0.03 MB. Copyright © 2019 Kothari et al. 2019 Kothari et al. This content is distributed under the terms of the Creative Commons Attribution 4.0 International license.

**FIG 7:**
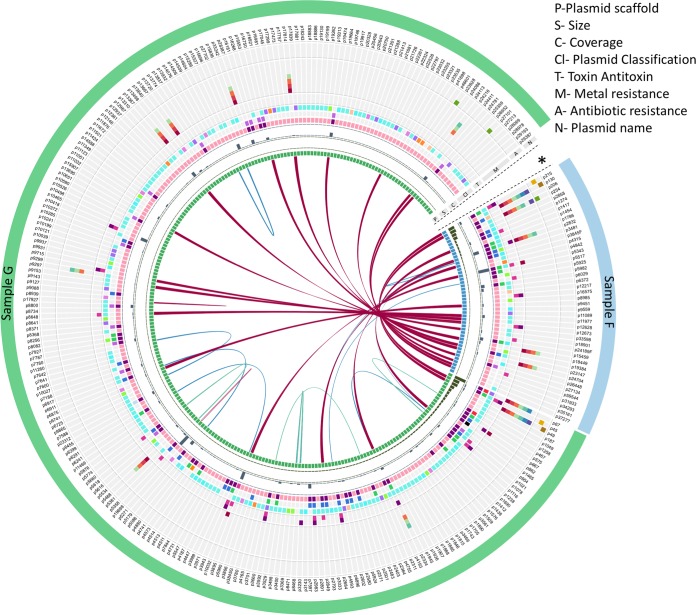
Visualization of circular plasmids of interest from the groundwater samples F (blue) and G (green). Cutoff values for blast are E value of e−5 and minimum match of 1,000 bp (97%> = magenta, 90%> = blue, and 80%> = teal). Rings 2 and 3 depict histograms corresponding to the scaffold size and sequence coverage, respectively. Ring 4 depicts plasmid mobility (the colors light pink, pink, and violet represent nonmobilizable, mobilizable, and conjugative, respectively), ring 5 depicts MOB type (the colors blue, green, purple, red, black, orange, and light blue represent MOBP, MOBQ, MOBV, MOBF, MOBC, MOBB, and MOBT, respectively), ring 6 depicts incompatibility groups (the colors teal, purple, blue, green, cream, pink, and orange represent IncA/C cgPMLST, IncA/C cgPMLST or IncA/C PMLST, IncF RST, Inc HI1 MLST, Inc HI2 DLST, IncI1 MLST, and IncN MLST, respectively), and rings 7 (pink) and 8 (purple) depict presence of toxin and antitoxin, respectively. Rings 9 to 15 depict presence of genes annotated (by KBase) to provide resistance to metals (the colors carmine, red, orange, light green, light blue, dark blue, and purple represent mercury, lead, cadmium, zinc, cobalt, copper, and arsenic, respectively), and rings 16 to 18 depict presence of genes annotated (by KBase) to provide resistance to antibiotics (the colors green, yellow, and brown represent acriflavine, polymyxin, and fosfomycin, respectively). The outermost ring depicts the plasmid name. The plot was created using Circos and Circoletto (64, 65). The asterisk indicates the circular plasmids not depicted on the figure because of lack of similarity with other plasmids and the lack of identifying features in rings 5 through 18.

**FIG 8:**
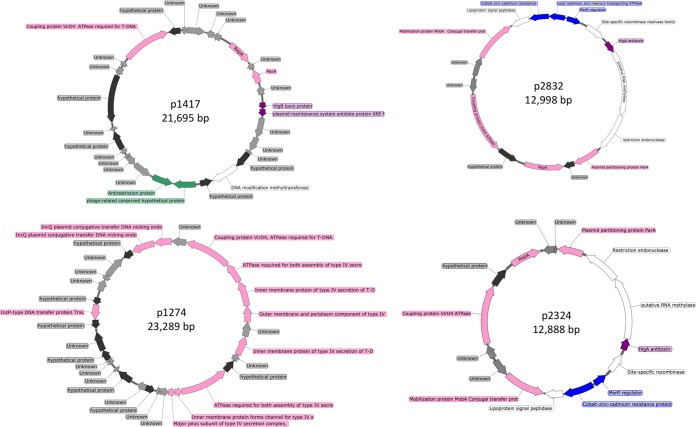
Circular plasmids common to both samples F and G. Genes carried are plasmid associated (pink), unknown (gray), hypothetical (black), metal resistance (blue), phage related (green), and toxin-antitoxin systems (purple).

To gain a comprehensive insight into the diversity of the plasmidome with potential similarity observed between groundwater samples, a subset of plasmids encoding features of interest were graphically compared for further analysis (Fig. 7). Overall, there was higher similarity between the two groundwater samples than within the sample itself. Of the circular plasmids from samples F and G, 18 plasmids shared almost the exact same sequence (plasmid maps of a selected few are depicted in Fig. 8; a detailed list is in Table S5c). One explanation for the plasmidome similarity between the samples could be the geographical proximity of the sampling sites and the fact that the groundwater flow is continuous and dynamic. Additionally, it may be that there are limited variations in the genetic modules that can constitute a plasmid. This is suggested by the presence of modules on the circular plasmids (e.g., plasmids p5343 and p67 described in this study) that show very high similarity to other plasmids reported from diverse geographical locations across the globe. Additional explanations include bias in plasmid DNA extraction methodology which might preferentially isolate plasmid DNA from certain bacterial subpopulations or the Phi29 amplification bias, which preferentially amplifies a subset of plasmids.

**FIG 9:**
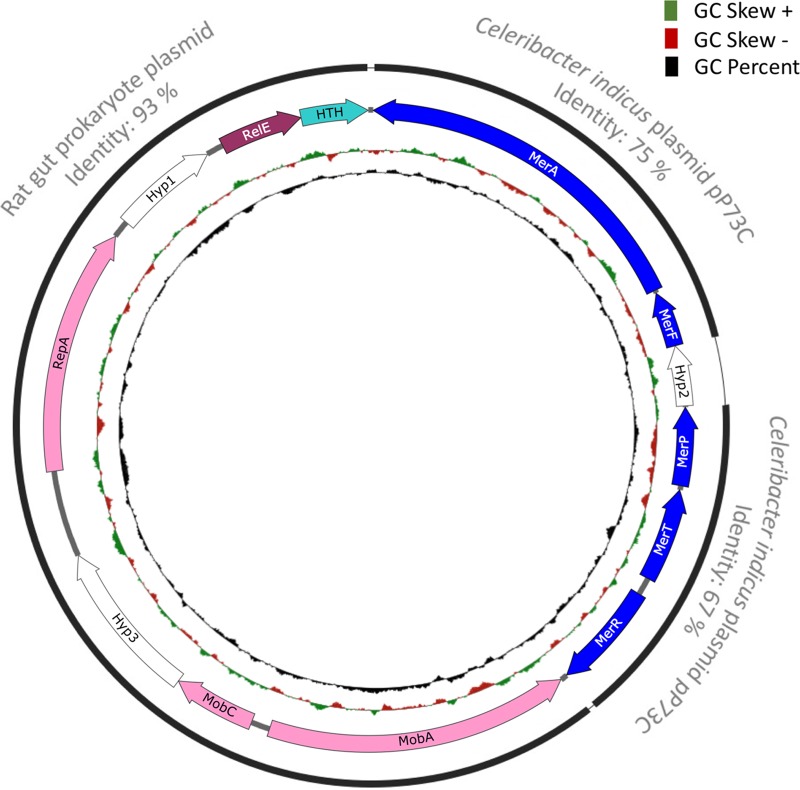
Plasmid map of p5343. Genes encode the following proteins: MerA, mercuric ion reductase; MerF, mercuric ion uptake protein; Hyp, hypothetical protein; MerP, mercuric transport protein; MerT, mercuric transport protein; MerR, regulator of mercury resistance genes; MobA, mobilization protein A; MobC, mobilization protein C; RepA, plasmid replication protein; HTH, helix-turn-helix domain protein; RelE, RelE toxin. The black lines indicate that the plasmid can be broken into different modules that show similarity to other previously reported plasmids (the closest NCBI BLAST hits with more than 92% query coverage are labeled in gray).

One of the most interesting plasmids identified was an 8-kb plasmid (p5343), highly abundant across samples, carrying genes annotated to be involved in mercury resistance along with plasmid mobilization and replication genes (Fig. 9). Most of the genes on this plasmid have homologs in the genus *Paracoccus.* The abundance of the genus *Paracoccus* was less than 0.5% based on 16S rRNA distribution, perhaps indicating that the plasmid might have consequently horizontally transferred into other hosts and/or is maintained in the original host in high copy numbers. Alternatively, it is possible that even at 0.5% *Paracoccus* is the primary genus in this environment that hosts the plasmid, given that not all bacteria host plasmids, and the numbers can be explained by plasmid DNA extraction and amplification biases along with a high copy number of the plasmid. Performing a nucleotide BLAST search reveals that this plasmid can be broken into three modules. The first module spans from *mobA* to the helix-turn-helix-containing protein and exhibits homology to a rat gut plasmid in GenBank, accession no. LN852881.1 (total size 12.9 kb). The original rat gut plasmid codes for certain hypothetical proteins in addition to the *ccdA/ccdB* type II toxin-antitoxin system genes. The second and third modules contain mercury resistance genes and depict homology to the native plasmid pP73c (total size, 122 kb) in Celeribacter indicus P73T. The P73T strain was isolated from a deep-sea sediment in the Indian Ocean (40). This study reveals that plasmids contain modules which are remarkably conserved in microbes in strikingly different environments, across the globe.

Intriguingly, even though mercury contamination is reported in certain sites nearby (31), these specific groundwater samples did not contain any detectable mercury, pointing to an interesting question—why do plasmids with metal resistance genes persist in such environments? Other circular plasmids also show the presence of genes annotated to provide mercury tolerance (Fig. 7), including a 28.5-kb plasmid, p667, which also contains the *mer* operon (Fig. S2). Plasmids are typically maintained when they confer a selective advantage to the host or replicate faster than the hosts. Besides, plasmid persistence could be attributed to compensatory adaption, along with brief periods of positive selection (41), which might be the most plausible explanation for the persistence of a metal resistance gene(s) on plasmids in the groundwater. Even then, the persistence over long periods might be linked to benefits of carrying the gene on a plasmid rather than in the chromosome, such as obtaining higher levels of expression. Our study suggests that the microbial community in groundwater is likely robust in tolerating low metal stresses and possesses a latent ability to swiftly adapt to changes in the environmental stress levels.

10.1128/mBio.02899-18.2 FIG S2 Plasmid map of p667 carrying mercury resistance (*mer*) operon. Genes carried are plasmid associated (pink), unknown (gray), hypothetical (black), metal resistance (blue), phage resistance (green), toxin-antitoxin systems (purple), and other annotated genes (yellow). Download FIG S2, DOCX file, 0.4 MB. Copyright © 2019 Kothari et al. 2019 Kothari et al. This content is distributed under the terms of the Creative Commons Attribution 4.0 International license.

### Conclusion.

This is the first study to explore the plasmidome of a groundwater environment based on metagenomic approaches. Given the low cell density and absence of selective parameters (e.g., mercury), along with the burden associated with carrying plasmids, it was surprising to find a rich plasmidome in the groundwater samples. Our study adds hundreds of novel plasmids to the plasmid database(s). Additionally, the optimized plasmid DNA isolation methods targeted large circular DNA molecules and identified the largest plasmids reported in plasmidome studies. Further, we find that plasmid distribution is more conserved across groundwater samples even though the microbiome fluctuates daily from well to well. In fact, our analyses also revealed the presence of certain identical plasmids from different groundwater samples. The predominance of genes encoding metal (including mercury) resistance on circular plasmids, despite the lack of detectable metals in the corresponding groundwaters, strongly implicates the native plasmids as the mechanism for maintaining latent functionalities in these environments. Interestingly, we find that antibiotic resistance genes are not as predominant as the metal resistance genes, indicating that a lack of selective pressure (i.e., no use of antibiotics) helps in curtailing the spread of antibiotic resistance. Together, the plasmidome analysis of this site provides a broad insight into plasmid-borne functions and provides evidence that plasmid-mediated horizontal gene transfer plays a role in driving the evolution of this groundwater microbial community. Although certain observations made were unique to this site, the method to examine native plasmid DNA in low-cell-density environments and the broad trends observed are generalizable to all microbial communities.

## MATERIALS AND METHODS

### Sample collection.

Water samples were collected from groundwater wells of the Department of Energy’s ORFRC, Tennessee (22) (well locations provided in Fig. S3 in the supplemental material). Given the difficulties in groundwater sampling, coupled with the fact that groundwater represents continuous dynamic water flow below the Earth’s surface, these samples serve as survey snapshots rather than replicates. Prior to collection of samples, approximately 5 to 20 liters of groundwater was pumped until temperature, pH, conductivity, and oxidation-reduction (redox) values were stabilized to purge the well and the line of standing groundwater. Bulk groundwater measurements and geochemical sample collections (21) were conducted (Table S7). For 16S rRNA analysis and plasmid DNA isolation, a total of 8 and 5 liters of water, respectively, was filtered through 10-μm and 0.2-μm nylon filters (Sterlitech Corporation, Kent, WA, USA). Filters were immediately stored on dry ice in 50-ml Falcon tubes until being transported to the −80°C freezer.

10.1128/mBio.02899-18.3 FIG S3 (a) List of samples in this study. (b and c) Plan-view maps of the ORFRC, showing large- (b) and small-scale (c) features. Download FIG S3, DOCX file, 3.2 MB. Copyright © 2019 Kothari et al. 2019 Kothari et al. This content is distributed under the terms of the Creative Commons Attribution 4.0 International license.

10.1128/mBio.02899-18.10 TABLE S7 Geochemical parameters measured. The following concentrations were below the detection limit: arsenic, cadmium, cobalt, cesium, lead, aluminum, uranium, silver, chromium, nickel, copper, selenium, phosphate, nitrate, bromide, and mercury. The centering material for the PVC groundwater pipe in the wells contained mercury only in rare instances. The value highlighted in gray was below the detection limit. AODC, acridine orange direct count; DIC, dissolved inorganic carbon; DOC, dissolved organic carbon; NA, data not available. Download Table S7, DOCX file, 0.01 MB. Copyright © 2019 Kothari et al. 2019 Kothari et al. This content is distributed under the terms of the Creative Commons Attribution 4.0 International license.

### Geochemical measurements.

Temperature, pH, conductivity, redox, and dissolved oxygen were measured at the wellhead using an In-Situ Troll 9500 (In-situ Inc., Fort Collins, CO, USA). Sulfide and ferrous ion groundwater concentrations were determined using the USEPA methylene blue method (Hach 8131) and 1,10-phenanthroline method (Hach 8146), respectively, and analyzed with a field spectrophotometer (Hach DR 2800). All other biological and geochemical parameters were measured as previously described (21). Mercury analysis was performed on samples containing 25 ml groundwater and 25 ml glycerol by oxidation, purge, trap, and cold vapor atomic fluorescence spectrometry 1631E at ALS Environmental, Kelso, WA, USA.

### Plasmid DNA isolation optimization.

A model system of a 1:1:1 mixture of Desulfovibrio vulgaris Hildenborough (ATCC 29579) containing a 202-kb native plasmid (pDV1), Escherichia coli DH1 (ATCC 33849) containing a 48-kb fosmid (fSCF#19) (42), and E. coli strain J-2561 containing a 5-kb (pBbS5c) plasmid was prepared using cells grown to an optical density (at 600 nm) of 1. *Desulfovibrio* was grown in LS4D supplemented with 0.1% (wt/vol) yeast extract (43) while E. coli was grown in LB medium. This mixture was serially diluted 10-fold; stored at −80°C; and used to test, compare, and optimize plasmid detection via quantitative PCR (qPCR). Two alkaline hydrolysis methods were compared to preferentially isolate plasmid DNA (44, 45). Residual linear chromosomal DNA fragments were minimized by plasmid-safe ATP-dependent DNase (Epicentre, Madison, WI, USA) treatment for 24 to 48 h at 37°C. The presence of chromosomal DNA was tested by PCR using 16S rRNA universal primers (BAC338F, 5′-ACTCCTACGGGAGGCAG-3′, and BAC805R, 5′-GACTACCAGGGTATCTAATCC-3′) (46). If 16S rRNA PCR product was visible on a 1% agarose gel, another overnight digestion reaction was performed until the product could no longer be visualized. The DNase was inactivated at 70°C for 30 min. The DNA was then amplified with Phi29 DNA polymerase (New England Biolabs, Ipswich, MA, USA) (16) at 4, 18, or 30°C for 168, 25, and 24 h, respectively. Plasmid DNA isolation was checked via qPCR against a specific plasmid-borne gene on all three plasmids. qPCR was performed using the SsoAdvanced Universal SYBR Green Supermix (Bio-Rad, Hercules, CA, USA) per the manufacturer’s protocol. Total DNA from *D. vulgaris* Hildenborough was used as a control for the 202-kb primers, and the plasmid DNA coding for pBbS5c was used as a control for the 5-Kb primers. Additionally, since our samples were essentially contained on filters, we tested whether the presence of filter interfered with plasmid DNA isolation. The filters were cut into smaller pieces and vortexed with beads in an attempt to improve plasmid recovery.

### Plasmid DNA isolation from environmental samples.

To extract DNA from bacteria on a filter, we modified an alkaline hydrolysis plasmid DNA isolation method (45) as described below. The filters from the groundwater samples A to E were thawed to room temperature, cut into pieces in a sterile petri dish using sterilized forceps and scissors, and split into two 50-ml Falcon tubes. The volumes of all reagents were multiplied 20 times to immerse each half-filter. Before the addition of lysozyme (Sigma-Aldrich, St. Louis, MO, USA), the samples were heated to 37°C with gentle inversion for 10 min and vortexed with 0.1-mm disrupter beads (Scientific Industries, Bohemia, NY, USA) at medium setting for 5 min. After the addition of sodium chloride, the liquid was transferred into 50-ml phase lock gel heavy tubes. A 14.5-ml amount of 25:24:1 phenol-chloroform-isoamyl alcohol was added to each tube, thoroughly mixed, and centrifuged for 5 min at 1,500 × *g* (Beckman Coulter Allegra 25R centrifuge). The upper phase was transferred to a fresh phase lock tube. A 14.5-ml amount of 24:1 chloroform-isoamyl alcohol was added and centrifuged for 5 min at 1,500 × g. The upper phase was transferred to a 50-ml Falcon tube and precipitated with an equal volume of isopropanol. The extractions from each half of the filter were recombined and incubated on ice for 1 h, followed by centrifugation for 5 min at 8,000 × *g*. The excess isopropanol was removed, and the pellet was resuspended in 1 ml of 10 mM Tris-1 mM EDTA, pH 7, transferred to a 1.6-ml tube, and dehydrated down to 50 µl with a Vacufuge Plus (Eppendorf; V-AQ, 45°C). The remnant linear DNA fragments were removed by plasmid-safe ATP-dependent DNase (Epicentre Biotechnologies, Madison, WI) at 37°C for 48 h with double the recommended ATP and enzyme amounts. The lack of chromosomal DNA contamination was confirmed by PCR with degenerate 16S rRNA primers. The plasmid DNA was amplified with Phi29 DNA polymerase (New England Biolabs, Ipswich, MA) as previously described (16) for 6 days at 18°C. This was followed by ethanol precipitation and use of a NanoDrop instrument to concentrate and quantify the DNA. For the plasmid DNA isolation from groundwater samples F and G, a variation to the method was that about 1.33 × 10^5^ cells of each plasmid-containing control strain were added to the filters to assess the efficiency of plasmid DNA isolation. The lack of chromosomal DNA contamination in plasmid DNA extracted from groundwater samples F and G was confirmed by PCR with degenerate 16S rRNA primers.

### Plasmid sequencing and bioinformatics.

For the groundwater samples A to E, the plasmid DNA libraries were pooled and sequenced on a single flow cell of the Illumina MiSeq reagent v3 kit (paired-end protocol), resulting in shallow sequencing. Plasmid DNA from two additional groundwater samples, F and G, was sequenced using the Illumina MiSeq reagent v3 kit (paired-end protocol) at the Vincent J. Coates Genomics Sequencing Laboratory at UC Berkeley (Berkeley, CA). An entire flow cell was used (no pooling of plasmid libraries), resulting in deep sequencing. The numbers of raw reads obtained by shallow and deep sequencing are depicted in Fig. 2. Trimmomatic 0.36 (http://www.usadellab.org/cms/?page=trimmomatic) was used to trim the reads with the following parameters: IlluminaClip:TruSeq3-PE.fa:2:30:10, Leading:3 Trailing:3 SlidingWindow:4:15 MinLen:36. As reported previously (17), IDBA-UD (47) was used for *de novo* read assembly with the parameter “–pre_correction.” Assembled sequences were searched against the SILVA 16S rRNA database (48) using BLASTN; all scaffolds with >200-bp identity to 16S rRNA were removed from further analysis. The proportion of reads that mapped to scaffolds with 16S rRNA coding genes was 0.65% and 1.07% for samples F and G, respectively. We removed the entire scaffolds and not just the reads mapping to 16S rRNA coding genes, to eliminate all potential chromosomal DNA contamination. To exclude the control plasmids in groundwater samples F and G, all sequences with more than 95% identity to these plasmids (minimum alignment length, 1,000 bp) were also removed. The resulting data set, referred to as “all_scaffolds,” was analyzed with the MG-RAST server (49) using similarity to the SEED database (with a maximum E value of ≤10^−5^) (50), generating taxonomic and functional assignments. All the sequence data generated are available via MG-RAST (IDs available in Fig. 2).

The sequencing coverage of plasmid DNA from groundwater samples F and G allowed additional analyses. We modified a pipeline method for postassembly detection of circularity among scaffolds (17) with the following criteria to identify the complete closed circular scaffolds referred to as “circular_scaffolds” or simply circular plasmids: (i) scaffold length of >2 kb, (ii) >34-bp homology (E value >1e−5) at the ends of the scaffold in the correct direction, and (iii) at least two read pairs mapping on opposite ends of the contig, a maximum of 500 bp from the end. The complete pipeline with Perl scripts can be found at https://github.com/yuwwu/detect-circ-plasmid. The “circular_scaffolds” were subjected to annotation using components from the RAST (Rapid Annotations using Subsystems Technology) toolkit (RASTtk) with the Department of Energy Systems Biology Knowledgebase, KBase (http://kbase.us) (51). Annotation of circular plasmids are available through https://narrative.kbase.us/narrative/ws.40055.obj.11.

The resulting “all_scaffolds” and “circular_scaffolds” plasmid sequences were compared with (i) A CLAssification of Mobile genetic Elements (ACLAME) (52, 53), (ii) the antiBacterial biocide and Metal resistance genes database (BacMet) (54), (iii) the Toxin Antitoxin DataBase (TADB) (55), (iv) the Antibiotic Resistance genes DataBase (ARDB) (56), and (v) the Comprehensive Antibiotic Resistance Database (CARD) (57). The analyses were performed as follows. (i) ACLAME plasmid proteins and MGE (Mobile Genetic Elements) families were downloaded from the ACLAME website. The plasmid genes from both samples were mapped against the plasmid proteins using BLAST with an E value cutoff of 1e−3. The BLAST tabulated results were parsed to obtain the taxonomic distributions of the plasmid genes by mapping the BLAST results to the MGE families, which consist of the taxonomic information. (ii) For BacMet, the Perl script BacMet-Scan.pl version 1.1, the predicted resistance gene data sets, and the experimentally confirmed resistance gene data set were downloaded from bacmet.biomedicine.gu.se. The BacMet-Scan.pl was executed using default parameters (-blast -e 1 -l 30 -p 90) to generate the tabulated report against both predicted and experimentally confirmed data sets. (iii) For TADB, the database was downloaded from the TADB website version 1.1 (http://202.120.12.135/TADB2/) followed by BLAST with the following parameters: E value of 1e−3, min_target_seqs 1. (iv) For ARDB, the Perl script ardbAnno.pl and ardbAnno.pm were downloaded from the ARDB website along with the resistance gene data set. The plasmid genes from both samples were mapped against the resistance gene data set using the scripts with default parameters. (v) For CARD, CARD and software RGI (Resistant Gene Identifier) databases were downloaded from the CARD website (https://card.mcmaster.ca/home). The script rgi.py was used to search the predicted plasmid genes against the CARD database with default parameters followed by parsing using a customized Perl script. The “circular_scaffolds” were also categorized into incompatibility groups (58) and the relaxase/MOB types (37).

### 16S rRNA gene sequencing.

Genomic DNA was extracted using the modified Miller DNA extraction method (21) followed by purification and concentration using a Genomic DNA Clean & Concentrator kit (Zymo Research, Irvine, CA). DNA quality was determined using the NanoDrop spectrophotometer (Thermo Scientific, Waltham, MA), and concentration was determined using a Qubit 2.0 fluorometer (Life Technologies, Carlsbad, CA). The V4 region of both bacterial and archaeal 16S rRNA genes was amplified using a two-step PCR approach. The primers (515F, 5′-GTGCCAGCMGCCGCGGTAA-3′, and 806R, 5′-GGACTACHVGGGTWTCTAAT-3′) were used without added sequencing components in the first step to avoid additional bias. To increase the base diversity in sequences of sample libraries, phasing primers were used in the second-step PCR. Spacers of different lengths (0 to 7 bases) were added before the forward and reverse primers, which shifts sequencing phases among different community samples from both directions. Sequencing was performed on the Illumina MiSeq platform (21).

The resulting 16S rRNA gene sequence data were processed using custom python scripts (https://github.com/almlab/SmileTrain) that call USEARCH for quality filtering and overlapping paired-end reads and Biopython (59) for file format input and output. The sequences were then progressively clustered to 90% with UCLUST (60), aligned to the SILVA database with mothur and align.seqs, and processed with distribution-based clustering as previously described (61) with k_fold 10 to remove sequencing errors. Chimeras were identified with UCHIME (62) and removed. Taxonomic identification was performed with the Ribosomal Database Project (63) using 0.50 as a confidence threshold for taxonomic classification at every level. The OTU table data were then converted to a biom format to analyze diversity and taxon summaries in Qiime.
