# Supplementary figures and images for: Large Circular Plasmids from Groundwater Plasmidomes Span Multiple Incompatibility Groups and Are Enriched in Multimetal Resistance Genes
Source: mBio. 2019 Feb 26;10(1):e02899-18. doi: 10.1128/mBio.02899-18 (PMC6391923; doi:10.1128/mBio.02899-18)

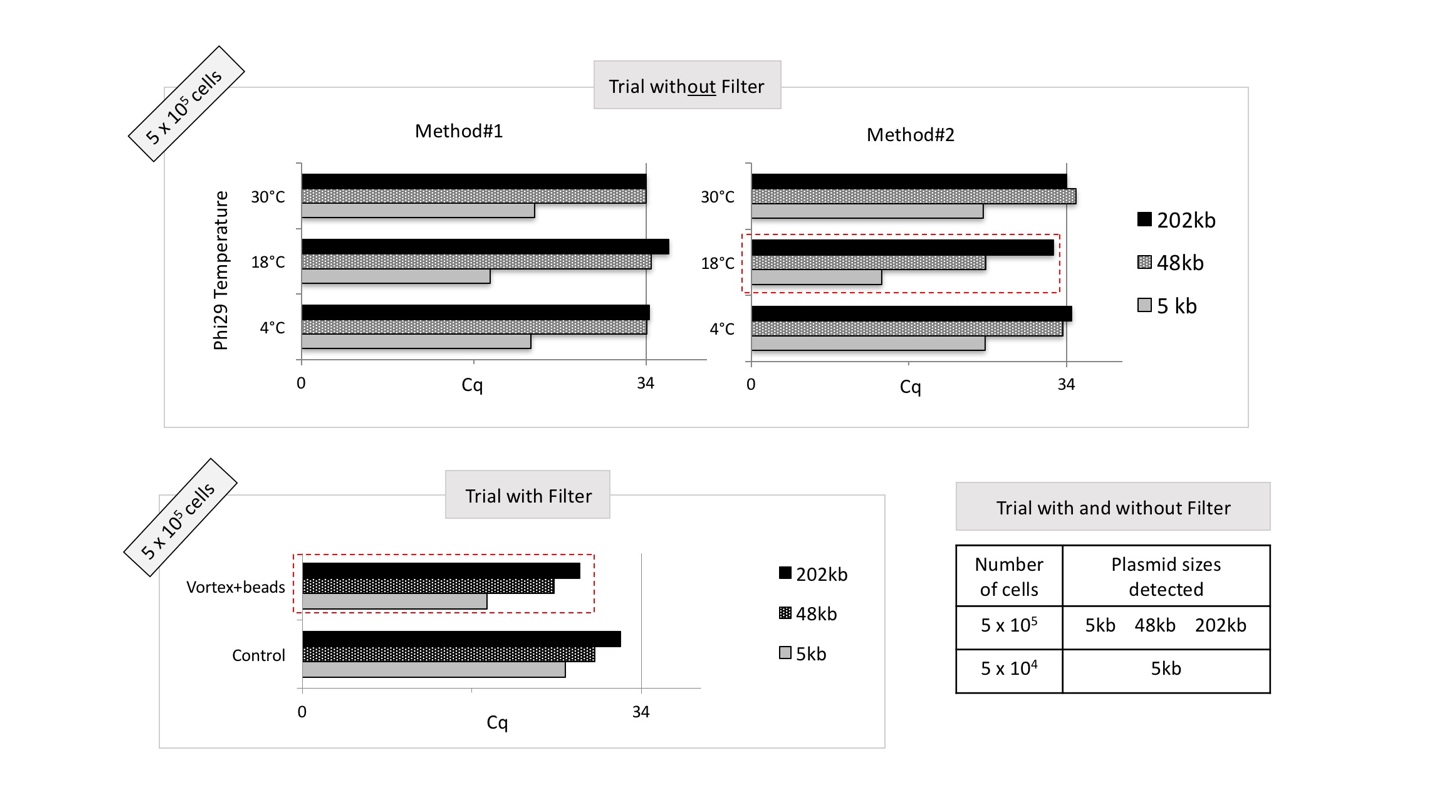


b.

a.

Supplement: FIG S1 [file mBio.02899-18-sf001.docx]

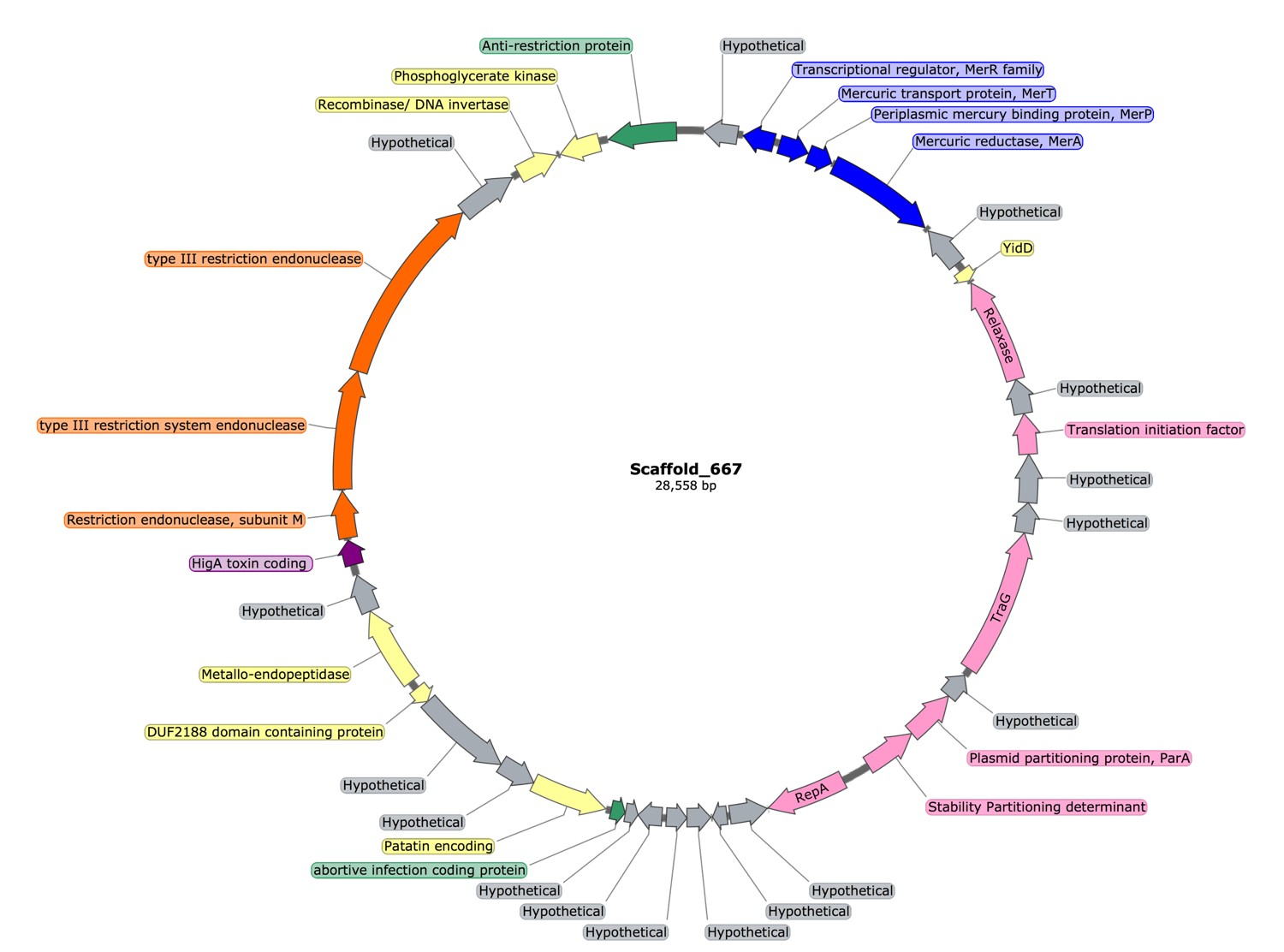

Supplement: FIG S2 [file mBio.02899-18-sf002.docx]
