# Supplementary material for: Large Circular Plasmids from Groundwater Plasmidomes Span Multiple Incompatibility Groups and Are Enriched in Multimetal Resistance Genes
Source: mBio. 2019 Feb 26;10(1):e02899-18. doi: 10.1128/mBio.02899-18 (PMC6391923; doi:10.1128/mBio.02899-18)
Supplement: TABLE S1 [file mBio.02899-18-st001.docx]

| **Category** | **Sample F** | | | **Sample G** |
| --- | --- | --- | --- | --- |
| *BacMet- Predicted genes database* | |  | |  |
| Gene annotation  – “all_scaffolds” | Total Hits: 487  heavy metal translocating P-type ATPase (12)  Arsenic resistance protein (12) | | | Total Hits: 646  Copper-translocating P-type ATPase (16)  LysR family transcriptional regulator (13) |
| Gene source  – “all_scaffolds” | Total Hits: 487  *Pseudomonas fluorescens* SBW25 (39)  *Pseudomonas fluorescens* WH6 (28) | | | Total Hits: 646  *Pseudomonas fluorescens* SBW25 (66)  *Pseudomonas fluorescens* Pf0-1 (62) |
| Resistance genes to compounds  – “all_scaffolds” | Total Hits: 487  Copper (66)  Triclosan (36)  Arsenic (33) | | | Total Hits: 646  Copper (99)  Triclosan (70)  Arsenic (34) |
| *BacMet - Experimentally confirmed genes database* | | |  |  |
| Gene annotation  – “all_scaffolds” | Total Hits: 162  Mercury reductase enzyme, merA (9)  Multidrug efflux RND transporter, MexF (4) | | | Total Hits: 192  Multidrug efflux RND transporter, permease protein MexF; part of mexE-mexF-oprN efflux operon (14)  Mercury reductase enzyme, merA (14) |
| Gene source  – “all_scaffolds” | Total Hits: 162  *Escherichia coli (strain K12) (80)*  *Pseudomonas stutzeri* (10) | | | Total Hits: 192  *Escherichia coli (strain K12) (84)*  *Pseudomonas fluorescens Pf-5/ATCC BAA-477* (20) |
| Resistance genes to compounds  – “all_scaffolds” | Total Hits: 162  Copper (23)  Mercury (17)  Triclosan (16) | | | Total Hits: 192  Copper (32)  Triclosan (28)  Mercury (20) |
| Abundant metal resistance genes plotted with the bacterial phyla the closest homolog is reported in. Edge thickness indicates the number of genes encoded. |  | | |  |
|  |  | | |  |
