# Supplementary material for: Large Circular Plasmids from Groundwater Plasmidomes Span Multiple Incompatibility Groups and Are Enriched in Multimetal Resistance Genes
Source: mBio. 2019 Feb 26;10(1):e02899-18. doi: 10.1128/mBio.02899-18 (PMC6391923; doi:10.1128/mBio.02899-18)
Supplement: TABLE S2 [file mBio.02899-18-st002.docx]

|  | Sample F | | | Sample G | |
| --- | --- | --- | --- | --- | --- |
| **ARDB-** Number of genes with hits | |  | 236 |  | 312 |
| Top categories | Bacitracin resistance  *bacA* | | 70.8 % | Bacitracin resistance  *bacA* | 42.9 % |
|  | Chlaramphenicol resistance *catA1* | | 2.5 % | Chlaramphenicol resistance *catA1* | 22.8 % |
|  | Nodulation cell division resistance *mexF* | | 2.5 % | Nodulation cell division resistance *mexF* | 4.8 % |
| **CARD-** Number of genes with hits | |  | 1002 |  | 862 |
| Top categories | Aminocoumarin resistance *alaS* | | 14.9 % | Aminocoumarin resistance *alaS* | 11.5 % |
|  | Elfamycin resistance | | 10.2 % | Elfamycin resistance | 7.8 % |
|  | mupirocin resistance *ileS* | | 7.7 % | DNA repair *mfd* | 7.1 % |
