# Supplementary material for: Large Circular Plasmids from Groundwater Plasmidomes Span Multiple Incompatibility Groups and Are Enriched in Multimetal Resistance Genes
Source: mBio. 2019 Feb 26;10(1):e02899-18. doi: 10.1128/mBio.02899-18 (PMC6391923; doi:10.1128/mBio.02899-18)
Supplement: TABLE S3 [file mBio.02899-18-st003.docx]

| **Category** | **Sample F** | **Sample G** |
| --- | --- | --- |
| Gene annotation  – “all_scaffolds” | Total Hits: 417828  hypothetical protein (79052)  ABC transporter related (3061) | Total Hits: 362921  hypothetical protein (70055)  ABC transporter related (2298) |
| Gene source  – “all_scaffolds” | Total Hits: 417828  *Ralstonia solanacearum* GMI1000 (31019)  Sinorhizobium meliloti 1021 (27117) | Total Hits: 362921  *Ralstonia solanacearum* GMI1000 (29419)  *Sinorhizobium meliloti* (23173) |
| Gene annotation  – “circular_scaffolds” | Total Hits: 406  hypothetical protein (121)  outer membrane efflux protein (7) | Total Hits: 1486  hypothetical protein (449)  Mobilization protein (24) |
| Gene source  – “circular_scaffolds” | Total Hits: 406  *Pelobacter propionicus* DSM 2379 (26)  *Verminephrobacter eiseniae EF01-2* (22) | Total Hits: 1486  *Sphingomonas wittichii* RW1 (66)  *Ralstonia solanacearum GMI1000 (47)* |
