# Supplementary material for: Large Circular Plasmids from Groundwater Plasmidomes Span Multiple Incompatibility Groups and Are Enriched in Multimetal Resistance Genes
Source: mBio. 2019 Feb 26;10(1):e02899-18. doi: 10.1128/mBio.02899-18 (PMC6391923; doi:10.1128/mBio.02899-18)
Supplement: TABLE S4 [file mBio.02899-18-st004.docx]

|  | Toxin (%) | Antitoxin (%) | Regulator (%) | Toxin and antitoxin (%) | Toxin, antitoxin, and regulator (%) |
| --- | --- | --- | --- | --- | --- |
| Sample F “all_scaffolds” | 2.1 | 2.0 | 0.3 | 0.8 | 0.1 |
| Sample G “all_scaffolds” | 1.6 | 1.6 | 0.3 | 0.6 | 0.1 |
| Sample F “circular_scaffolds” | 23.5 | 29.4 | 7.4 | 19.1 | 4.4 |
| Sample G “circular_scaffolds” | 10.4 | 13.1 | 3.3 | 7.7 | 1.8 |
