# Supplementary material for: Large Circular Plasmids from Groundwater Plasmidomes Span Multiple Incompatibility Groups and Are Enriched in Multimetal Resistance Genes
Source: mBio. 2019 Feb 26;10(1):e02899-18. doi: 10.1128/mBio.02899-18 (PMC6391923; doi:10.1128/mBio.02899-18)
Supplement: TABLE S5 [file mBio.02899-18-st005.docx]

| **Table a)** |  |  |  |  |
| --- | --- | --- | --- | --- |
| **Sample F plasmid** | **Coverage** | **Plasmid size (kb)** | **Resistance Genes** | **Plasmid associated genes** |
| p5343 | 869 | 7993 | Metal Resistance (Mercury) | Mobilization protein-coding gene *mobA, mobC*,  Replication protein-coding gene *repA* |
| p2832 | 445 | 12888 | Metal Resistance (Cobalt Zinc, Cadmium) | Plasmid partition protein-coding gene *parA*,  Antitoxin-coding gene *higA*,  Replication protein-coding gene *repA*,  Mobilization protein-coding gene *mobA* |
| p6029 | 288 | 7364 | - | Mobilization protein-coding gene |
| p15459 | 248 | 3832 | - | Mobilization protein-coding gene *mobA* |
| p11977 | 196 | 4554 | - | Doc Toxin-coding gene |
| p6372 | 186 | 7077 | - | - |
| p215 | 139 | 96159 | - | Antitoxin-coding gene *higA*,  Mobilization protein-coding gene *mobA*,  RelE/StbE replicon stabilization toxin-coding gene,  RelB/StbD replicon stabilization protein-coding gene (antitoxin to RelE/StbE),  IncF plasmid conjugative transfer pilus assembly protein-coding genes *traH, traC, traC, traB*  Plasmid partition protein-coding gene *parA* |
| p1417 | 105 | 21696 | - | Replication protein-coding gene *repA*,  Plasmid partition protein-coding gene *parA*,  Toxin-coding gene *higB*,  plasmid maintenance system antidote protein - XRE family,  Coupling protein-coding gene *virD4* |
| p9451 | 102 | 5372 | - | - |
| p117 | 84 | 287339 | Phage resistance protein | - |
| p24704 | 74 | 2796 | Metal Resistance (Zinc) | - |
| p18901 | 73 | 3916 | - | - |
| p12628 | 35 | 4388 | - | - |
| p35161 | 29 | 2218 | - | - |
| p12673 | 28 | 4335 | No | Toxin-coding gene *yoeB*  Antitoxin-coding gene *yefM* |
| p130 | 19 | 94434 | Metal Resistance (Cobalt Zinc, Cadmium, Copper, Arsenic),  Antibiotic Resistance (Fosfomycin,  Spectinomycin),  Metal uptake (Potassium uptake) | - |
| p3845 | 17 | 10328 | - | - |
| p34293 | 10 | 2253 | - | - |
| p23147 | 8 | 2916 | - | - |
| p31933 | 7 | 2363 | - | - |
| p27134 | 6 | 2632 | - | - |

| **Table b)** | |  | |  |  | | |  | | |
| --- | --- | --- | --- | --- | --- | --- | --- | --- | --- | --- |
| **Sample G plasmid** | | **Coverage** | | **Plasmid size (kb)** | **Resistance Genes** | | | **Plasmid associated genes** | | |
| p23986 | | 2593 | | 2300 | - | | | - | | |
| p28338 | | 1799 | | 2059 | - | | | - | | |
| p8056 | | 910 | | 4782 | - | | | - | | |
| p19698 | | 890 | | 6427 | - | | | - | | |
| p10032 | | 617 | | 7993 | Metal Resistance (Mercury) | | | Mobilization protein-coding genes *mobA, mobC*,  Replication protein-coding gene *repA* | | |
| p10418 | | 542 | | 4017 | - | | | RelB/StbD replicon stabilization protein-coding gene (antitoxin to RelE/StbE),  RelE/StbE replicon stabilization toxin-coding gene | | |
| p15696 | | 424 | | 3049 | - | | | - | | |
| p13367 | | 379 | | 3388 | - | | | - | | |
| p7597 | | 357 | | 9688 | - | | | - | | |
| p28387 | | 325 | | 3015 | - | | | - | | |
| p19474 | | 325 | | 2632 | - | | | Replication protein-coding gene | | |
| p5179 | | 312 | | 6544 |  | | | Mobilization protein-coding genes *mobC* | | |
| p24781 | | 298 | | 2253 | - | | | - | | |
| p25039 | | 252 | | 2238 | - | | | - | | |
| p8800 | | 210 | | 4498 | - | | | - | | |
| p16750 | | 191 | | 2916 | - | | | - | | |
| p2324 | | 171 | | 12888 | Metal Resistance (Cobalt Zinc, Cadmium, Mercury) | | | Plasmid partition protein-coding gene *parA*,  Replication protein-coding gene *repA,*  Mobilization protein-coding genes *mobA,*  Coupling protein-coding gene *virD4* | | |
| p23312 | | 154 | | 5574 | - | | | Mobile element protein,  Integrase | | |
| p3529 | | 149 | | 8684 | - | | | Coupling protein-coding gene *virD4,*  Mobilization protein-coding genes *mobA* | | |
|  | |  | |  |  |  |  |  |  |  |
| **Table c)** |  | |  | |  |  |  | |  |  |
| **Sample F plasmid** | **Coverage** | | **Sample G plasmid** | | **Coverage** | **Mobility** | **Plasmid size (bp)** | | **Resistance Genes** | **Plasmid associated genes** |
| p5343 | 869 | | p10032 | | 617 | Mobilizable | 7993 | | Metal Resistance (Mercury) | Mobilization protein-coding gene *mobA*,  *mobC*,  Replication protein-coding gene *repA* |
| p2832 | 445 | | p2324 | | 171 | Conjugative | 12888 | | Metal Resistance (Cobalt Zinc, Cadmium) | Plasmid partition protein-coding gene *parA*,  Antitoxin-coding gene *higA*,  Replication protein-coding gene *repA,* Mobilization protein-coding gene *mobA* |
| p11977 | 196 | | p8648 | | 64 | Non- Mobilizable | 4554 | | - | Doc Toxin-coding gene |
| p215 | 139 | | p45 | | 79 | Conjugative | 96159 | | - | Antitoxin-coding gene *higA*,  Mobilization protein-coding gene *mobA*,  RelE/StbE replicon stabilization toxin, RelB/StbD replicon stabilization protein-coding gene (antitoxin to RelE/StbE),  IncF plasmid conjugative transfer pilus assembly protein-coding genes *traH, traC, traC, traB*,  Plasmid partition protein-coding gene *parA* |
| p1417 | 105 | | p1021 | | 11 | Conjugative | 21696 | | - | Replication protein-coding gene *repA,* Plasmid partition protein-coding gene *parA*,  Toxin-coding gene *higB*,  plasmid maintenance system antidote protein - XRE family,  Coupling protein-coding gene *virD4* |
| p9451 | 102 | | p6875 | | 63 | Non- Mobilizable | 5372 | | - | - |
| p18449 | 83 | | p13367 | | 379 | Non- Mobilizable | 3388 | | - | - |
| p24704 | 74 | | p17814 | | 52 | Non- Mobilizable | 2796 | | Metal Resistance (Zinc) | - |
| p18901 | 73 | | p13695 | | 25 | Non- Mobilizable | 3916 | | - | - |
| p12628 | 35 | | p9153 | | 37 | Non- Mobilizable | 4388 | | - | - |
| p35161 | 29 | | p25359 | | 47 | Non- Mobilizable | 2218 | | - | - |
| p12673 | 28 | | p9299 | | 17 | Non- Mobilizable | 4335 | | - | Toxin-coding gene *yoeB,*  Antitoxin-coding gene *yefM* |
| p130 | 19 | | p49 | | 88 | Non- Mobilizable | 94434 | | Metal Resistance (Cobalt Zinc, Cadmium, Copper, Arsenic),  Antibiotic Resistance (Fosfomycin,  Spectinomycin),  Metal uptake (Potassium uptake) | - |
| p3845 | 17 | | p2793 | | 17 | Conjugative | 10328 | | - | - |
| p34293 | 10 | | p24781 | | 298 | Non- Mobilizable | 2253 | | - | - |
| p23147 | 8 | | p16750 | | 191 | Conjugative | 2916 | | - | - |
| p31933 | 7 | | p23032 | | 12 | Non- Mobilizable | 2363 | | - | - |
| p27134 | 6 | | p19474 | | 325 | Conjugative | 2632 | | - | - |
