# Supplementary material for: Large Circular Plasmids from Groundwater Plasmidomes Span Multiple Incompatibility Groups and Are Enriched in Multimetal Resistance Genes
Source: mBio. 2019 Feb 26;10(1):e02899-18. doi: 10.1128/mBio.02899-18 (PMC6391923; doi:10.1128/mBio.02899-18)
Supplement: TABLE S6 [file mBio.02899-18-st006.docx]

| **Start** | **Strand** | **Length** | **Function** |
| --- | --- | --- | --- |
| 1 | + | 303 | hypothetical protein |
| 476 | - | 177 | - |
| 518 | + | 270 | Integration host factor alpha subunit |
| 841 | + | 627 | Mll7390 protein |
| 2799 | - | 1323 | hypothetical protein |
| 3473 | - | 675 | response regulator in two-component regulatory system with PhoQ |
| 4864 | - | 1383 | hypothetical protein |
| 8025 | - | 3165 | Cobalt-zinc-cadmium resistance protein CzcA; Cation efflux system protein CusA |
| 9029 | - | 1005 | hypothetical protein |
| 9157 | + | 612 | hypothetical protein |
| 9909 | + | 171 | - |
| 10093 | + | 1056 | Chloride channel protein |
| 11208 | + | 654 | Chloride channel protein |
| 12091 | - | 210 | - |
| 13090 | - | 831 | Protein-L-isoaspartate O-methyltransferase (EC 2.1.1.77) |
| 14118 | - | 1032 | hypothetical protein |
| 14598 | - | 456 | hypothetical protein |
| 17666 | - | 3072 | Cobalt-zinc-cadmium resistance protein CzcA; Cation efflux system protein CusA |
| 18691 | - | 1026 | hypothetical protein |
| 20013 | - | 1326 | Heavy metal RND efflux outer membrane protein, CzcC family |
| 20387 | - | 318 | Propeptide PepSY amd peptidase M4 |
| 20534 | + | 672 | response regulator in two-component regulatory system with PhoQ |
| 21202 | + | 1371 | Sensory histidine protein kinase |
| 22578 | + | 525 | hypothetical protein |
| 24059 | + | 2346 | hypothetical protein |
| 27377 | - | 1002 | Putative phospholipase C |
| 28282 | + | 771 | hypothetical protein |
| 29040 | + | 1179 | - |
| 30257 | + | 933 | hypothetical protein |
| 31186 | + | 513 | hypothetical protein |
| 31709 | + | 1188 | group 1 glycosyl transferase |
| 32899 | + | 171 | - |
| 33066 | + | 1074 | - |
| 34519 | + | 1008 | - |
| 35532 | + | 681 | - |
| 36205 | + | 1347 | hypothetical protein |
| 38304 | - | 702 | hypothetical protein |
| 40655 | - | 2352 | hypothetical protein |
| 41249 | - | 453 | - |
| 41982 | - | 693 | protein of unknown function DUF125, transmembrane |
| 42503 | - | 525 | - |
| 42980 | + | 558 | - |
| 44302 | - | 657 | Non-specific DNA-binding protein Dps / Iron-binding ferritin-like antioxidant protein / Ferroxidase (EC 1.16.3.1) |
| 45776 | - | 1356 | hypothetical protein |
| 46440 | - | 678 | response regulator in two-component regulatory system with PhoQ |
| 46573 | + | 1266 | Heavy metal RND efflux outer membrane protein, CzcC family |
| 47835 | + | 3180 | Cobalt-zinc-cadmium resistance protein CzcA; Cation efflux system protein CusA |
| 51021 | + | 897 | - |
| 53155 | - | 1047 | hypothetical protein |
| 53389 | + | 243 | - |
| 53628 | + | 390 | hypothetical protein |
| 54190 | + | 1281 | - |
| 55467 | + | 516 | hypothetical protein |
| 55979 | + | 1953 | hypothetical protein |
| 58273 | + | 528 | - |
| 58801 | + | 612 | hypothetical protein |
| 59449 | + | 444 | - |
| 63330 | - | 3231 | Cobalt-zinc-cadmium resistance protein CzcA; Cation efflux system protein CusA |
| 64496 | - | 1170 | Cobalt/zinc/cadmium efflux RND transporter, membrane fusion protein, CzcB family |
| 65752 | - | 1260 | Heavy metal RND efflux outer membrane protein, CzcC family |
| 67512 | - | 1326 | Probable NreB protein |
| 67600 | + | 270 | NreA-like protein |
| 68418 | - | 384 | Heavy metal resistance transcriptional regulator HmrR |
| 70889 | - | 2475 | Lead, cadmium, zinc and mercury transporting ATPase (EC 3.6.3.3) (EC 3.6.3.5); Copper-translocating P-type ATPase (EC 3.6.3.4) |
| 71942 | - | 930 | Copper resistance protein D |
| 72315 | - | 372 | copper resistance protein CopC |
| 72439 | + | 273 | - |
| 73145 | + | 576 | sigma-24 (FecI-like) protein |
| 73781 | + | 1938 | Multicopper oxidase |
| 75715 | + | 1212 | Copper resistance protein B |
| 76917 | + | 753 | - |
| 77707 | + | 390 | Copper resistance protein B |
| 78152 | + | 444 | CopG protein |
| 78836 | - | 210 | - |
| 79272 | - | 396 | - |
| 80201 | - | 939 | Cobalt-zinc-cadmium resistance protein CzcD |
| 80484 | - | 255 | Putative cytoplasmic protein |
| 80757 | + | 1458 | Beta-lactamase class C and other penicillin binding proteins |
| 82948 | - | 222 | - |
| 83655 | - | 273 | - |
| 84008 | - | 354 | hypothetical protein |
| 84452 | - | 318 | - |
| 85126 | - | 678 | - |
| 85427 | + | 582 | hypothetical protein |
| 86918 | - | 378 | - |
| 87761 | - | 816 | - |
| 88561 | - | 744 | - |
| 88853 | - | 303 | - |
| 90055 | - | 1188 | - |
| 90847 | - | 762 | hypothetical protein |
| 94113 | - | 3264 | IncW plasmid conjugative relaxase protein TrwC (TraI homolog) |
| 96449 | - | 2334 | IncW plasmid conjugative protein TrwB (TraD homolog) |
| 96726 | - | 288 | - |
| 97157 | - | 168 | - |
| 97272 | + | 225 | - |
| 98253 | - | 582 | - |
| 98983 | - | 342 | - |
| 100670 | - | 1089 | hypothetical protein |
| 101194 | - | 447 | hypothetical protein |
| 102209 | - | 138 | - |
| 102263 | + | 438 | - |
| 103691 | - | 432 | hypothetical protein |
| 103939 | - | 249 | - |
| 104078 | + | 489 | - |
| 104865 | - | 285 | - |
| 105549 | - | 540 | - |
| 106133 | - | 588 | - |
| 106423 | - | 294 | hypothetical protein |
| 106635 | - | 216 | - |
| 107011 | - | 375 | - |
| 107310 | - | 279 | hypothetical protein |
| 107495 | + | 1413 | hypothetical protein |
| 108910 | + | 1773 | hypothetical protein |
| 110685 | + | 2109 | diguanylate cyclase/phosphodiesterase (GGDEF & EAL domains) with PAS/PAC sensor(s) |
| 112790 | + | 369 | hypothetical protein |
| 113889 | - | 405 | Transcriptional regulator, MerR family |
| 113954 | + | 657 | Cobalt-zinc-cadmium resistance protein CzcD |
| 116559 | - | 1872 | TonB-dependent receptor; Outer membrane receptor for ferrienterochelin and colicins |
| 117100 | + | 1551 | Transposase |
| 118662 | + | 315 | Mobile element protein |
| 119006 | + | 354 | Mobile element protein |
| 119504 | + | 330 | Ethidium bromide-methyl viologen resistance protein EmrE |
| 120768 | - | 660 | hypothetical protein |
| 122443 | - | 1674 | hypothetical protein |
| 123878 | - | 1293 | hypothetical protein |
| 124370 | - | 417 | hypothetical protein |
| 125825 | - | 1410 | hypothetical protein |
| 126397 | - | 543 | hypothetical protein |
| 126743 | - | 312 | - |
| 127059 | - | 297 | - |
| 127717 | - | 645 | hypothetical protein |
| 127951 | - | 132 | - |
| 128640 | - | 693 | - |
| 129137 | - | 534 | hypothetical protein |
| 129334 | - | 207 | - |
| 129969 | - | 639 | - |
| 130893 | - | 909 | hypothetical protein |
| 133227 | - | 2238 | hypothetical protein |
| 137561 | - | 4218 | hypothetical protein |
| 138453 | - | 855 | hypothetical protein |
| 138822 | - | 357 | hypothetical protein |
| 138919 | + | 270 | - |
| 140485 | - | 1293 | hypothetical protein |
| 141595 | + | 402 | Mobile element protein |
| 141993 | + | 843 | Mobile element protein |
| 143801 | - | 789 | hypothetical protein |
| 143816 | + | 246 | - |
| 145112 | - | 888 | RepA |
| 145658 | - | 261 | - |
| 146275 | - | 507 | - |
| 146902 | - | 513 | - |
| 147256 | + | 321 | Conjugative transfer protein TraA |
| 147636 | + | 291 | IncF plasmid conjugative transfer pilus assembly protein TraL |
| 147928 | + | 573 | IncF plasmid conjugative transfer pilus assembly protein TraE |
| 148497 | + | 822 | IncF plasmid conjugative transfer pilus assembly protein TraK |
| 149322 | + | 1338 | IncF plasmid conjugative transfer pilus assembly protein TraB |
| 150646 | + | 879 | Thiol:disulfide involved in conjugative transfer |
| 151521 | + | 957 | hypothetical protein |
| 152474 | + | 2583 | IncF plasmid conjugative transfer pilus assembly protein TraC |
| 155062 | + | 342 | - |
| 155400 | + | 657 | Conjugative transfer protein ELI_00880 |
| 156585 | + | 639 | IncF plasmid conjugative transfer pilus assembly protein TraW |
| 157223 | + | 279 | hypothetical protein |
| 157498 | + | 927 | IncF plasmid conjugative transfer pilus assembly protein TraU |
| 158493 | + | 741 | IncF plasmid conjugative transfer protein TrbC |
| 159234 | + | 1794 | IncF plasmid conjugative transfer protein TraN |
| 161393 | + | 927 | IncF plasmid conjugative transfer pilus assembly protein TraF |
| 162319 | + | 1461 | IncF plasmid conjugative transfer pilus assembly protein TraH |
| 163792 | + | 2769 | IncF plasmid conjugative transfer protein TraG |
| 166778 | - | 216 | - |
| 166838 | + | 381 | - |
| 168114 | - | 288 | - |
| 171382 | - | 3264 | DNA polymerase III alpha subunit (EC 2.7.7.7) |
| 173842 | - | 765 | - |
| 173903 | + | 375 | - |
| 447 | - | 354 | - |
| 1174 | - | 669 | 2-iminoacetate synthase (ThiH) (EC 4.1.99.19) |
| 100 | + | 432 | Bll3346 protein |
| 1173 | - | 234 | - |
| 2 | + | 192 | - |
| 915 | - | 432 | - |
| 925 | + | 249 | - |
| 136 | - | 135 | - |
| 508 | - | 363 | - |
| 807 | - | 303 | hypothetical protein |
| 1173 | - | 180 | - |
| 1173 | - | 1173 | hypothetical protein |
| 304 | + | 606 | hypothetical protein |
| 3 | + | 1170 | Adenylate cyclase (EC 4.6.1.1) |
| 2 | + | 564 | serine protease |
| 817 | + | 354 | serine protease |
| 265 | - | 264 | ABC transporter, permease protein |
| 1 | + | 462 | - |
| 1327 | + | 1050 | Quinolinate synthetase (EC 2.5.1.72) |
| 3402 | - | 993 | Phytanoyl-CoA dioxygenase |
| 3417 | + | 675 | - |
| 4093 | + | 1011 | Metallo-beta-lactamase family protein |
| 5085 | + | 360 | - |
| 2 | + | 885 | - |
| 1169 | - | 1167 | - |
| 1170 | - | 450 | RNA polymerase ECF-type sigma factor |
| 296 | - | 294 | hypothetical protein |
| 1168 | - | 876 | Cysteine desulfurase (EC 2.8.1.7), NifS subfamily |
| 164 | + | 819 | Zn-dependent aminopeptidase |
| 101 | - | 99 | - |
| 456 | + | 207 | - |
| 907 | + | 255 | - |
| 1387 | + | 1410 | - |
| 3587 | - | 435 | - |
| 3676 | + | 921 | - |
| 4603 | + | 360 | - |
| 4959 | + | 300 | - |
| 1 | + | 438 | Ubiquinol--cytochrome c reductase, cytochrome B subunit (EC 1.10.2.2) |
| 445 | + | 567 | Ubiquinol-cytochrome C reductase iron-sulfur subunit (EC 1.10.2.2) |
| 1023 | + | 144 | Ubiquinol--cytochrome c reductase, cytochrome B subunit (EC 1.10.2.2) |
| 2 | + | 120 | - |
| 1166 | - | 186 | Peptidyl-prolyl cis-trans isomerase PpiD (EC 5.2.1.8) |
| 723 | + | 216 | - |
| 2 | + | 393 | tRNA pseudouridine synthase A (EC 4.2.1.70) |
| 354 | + | 813 | Phosphoribosylanthranilate isomerase (EC 5.3.1.24) |
| 3 | + | 1149 | adenine specific DNA methyltransferase |
| 1164 | - | 1164 | hypothetical protein |
| 23340 | - | 216 | - |
| 23383 | + | 717 | - |
| 27615 | - | 162 | - |
| 27641 | + | 639 | - |
| 34185 | + | 120 | - |
| 42858 | + | 123 | - |
| 65778 | + | 174 | - |
| 66005 | + | 120 | - |
| 72831 | + | 318 | hypothetical protein |
| 82572 | + | 120 | - |
| 82501 | - | 222 | - |
| 86311 | - | 246 | - |
| 98562 | - | 147 | - |
| 98985 | + | 252 | - |
| 101426 | + | 138 | - |
| 101732 | - | 135 | - |
| 103263 | - | 120 | - |
| 140672 | - | 174 | - |
| 140720 | + | 783 | hypothetical protein |
| 156076 | + | 513 | Conjugative signal peptidase TrhF |
| 161029 | + | 384 | hypothetical protein |
| 167662 | - | 147 | - |
| 173061 | - | 1683 | DNA polymerase-like protein PA0670 |
| 808 | + | 129 | - |
| 731 | - | 132 | - |
| 1127 | - | 159 | - |
| 717 | - | 195 | - |
| 1381 | + | 153 | - |
| 2055 | - | 159 | - |
| 2178 | + | 165 | - |
| 2699 | + | 123 | - |
| 3306 | - | 114 | - |
| 3844 | - | 123 | - |
| 4610 | - | 147 | hypothetical protein |
| 4789 | - | 129 | - |
| 4790 | + | 156 | - |
| 559 | - | 219 | - |
| 1118 | - | 513 | Cysteinyl-tRNA synthetase (EC 6.1.1.16) |
| 475 | + | 822 | hypothetical protein |
| 1163 | - | 156 | - |
| 321 | - | 129 | - |
| 333 | + | 168 | - |
| 185 | - | 162 | hypothetical protein |
| 263 | + | 171 | - |
| 4497 | + | 299 | repeat region |
| 932 | - | 76 | tRNA-His-GTG |
