# Supplementary material for: Large Circular Plasmids from Groundwater Plasmidomes Span Multiple Incompatibility Groups and Are Enriched in Multimetal Resistance Genes
Source: mBio. 2019 Feb 26;10(1):e02899-18. doi: 10.1128/mBio.02899-18 (PMC6391923; doi:10.1128/mBio.02899-18)
Supplement: TABLE S7 [file mBio.02899-18-st007.docx]

|  | **Sample A** | **Sample B** | **Sample C** | **Sample D** | **Sample E** | **Sample F** | **Sample G** |
| --- | --- | --- | --- | --- | --- | --- | --- |
| Temperature (°C) | 15.60 | 15.32 | 15.57 | 14.71 | 15.60 | 20.03 | 14.71 |
| AODC cells/mL | 172000.00 | 35200.00 | 1420.00 | 17013.23 | 172000.00 | 49858.22 | 17013.23 |
| Dissolved Oxygen (mg/L) | 1.23 | 1.74 | 1.41 | 0.11 | 1.23 | 0.51 | 0.11 |
| Conductivity (μS/cm) | 49.20 | 307.90 | 102.00 | 258.50 | 49.20 | 232.90 | 258.50 |
| Redox (mV) | 268.00 | 79.00 | 224.00 | -40.00 | 268.00 | -43.00 | -40.00 |
| pH | 4.50 | 6.60 | 5.01 | 7.46 | 4.50 | 7.98 | 7.46 |
| Sulfide (ppm) | 0.00 | 0.00 | 0.01 | 0.01 | 0.00 | 0.01 | 0.01 |
| Ferrous (mg/L) | 0.07 | 0.03 | 0.04 | 0.02 | 0.07 | 0.04 | 0.02 |
| DIC mg/L | 20.23 | 50.35 | 26.70 | 38.63 | 20.23 | 29.93 | 38.63 |
| DOC mg/L | 0.21 | 0.22 | 0.25 | 0.72 | 0.21 | 0.28 | 0.72 |
| Chloride mg/l | 1.06 | 1.17 | 1.20 | 0.87 | 1.06 | 0.80 | 0.87 |
| Sulfate mg/l | 7.72 | 5.77 | 5.84 | 7.41 | 7.72 | 6.11 | 7.41 |
| Barium mg/l | 0.05 | 0.08 | 0.04 | 0.58 | 0.05 | 0.58 | 0.58 |
| Calcium mg/l | 5.92 | 64.58 | 17.38 | 31.58 | 5.92 | 26.68 | 31.58 |
| Gallium mg/l | 0.00 | 0.00 | 0.00 | 0.02 | 0.00 | 0.02 | 0.02 |
| Potassium mg/l | 0.81 | 0.87 | 0.69 | 2.99 | 0.81 | 3.53 | 2.99 |
| Magnesium mg/l | NA | 1.73 | NA | 11.33 | NA | 10.99 | 11.33 |
| Manganese mg/l | 0.03 | 0.02 | 0.02 | 0.06 | 0.03 | 0.02 | 0.06 |
| Sodium mg/l | 0.71 | 1.30 | 0.79 | 14.14 | 0.71 | 34.77 | 14.14 |
| Strontium mg/l | 0.01 | 0.12 | 0.03 | 1.08 | 0.01 | 1.10 | 1.08 |
| Mercury | NA | NA | NA | 0.00 | NA | 0.00 | 0.00 |
| Zinc mg/l | 0.02 | 0.02 | 0.02 | 0.01 | 0.02 | 0.02 | 0.01 |
