# Supplementary material for: Large Circular Plasmids from Groundwater Plasmidomes Span Multiple Incompatibility Groups and Are Enriched in Multimetal Resistance Genes
Source: mBio. 2019 Feb 26;10(1):e02899-18. doi: 10.1128/mBio.02899-18 (PMC6391923; doi:10.1128/mBio.02899-18)

a

Sample Name	Well	Sampling Date	Filter Size (μm)
**Sample_A**	FW301	12/1/14	0.2
**Sample_B**	FW300	11/11/14	10
**Sample_C**	FW300	12/1/14	10
**Sample_D**	GW460	12/1/14	0.2
**Sample_E**	FW301	12/1/14	10
**Sample_F**	GW456	11/11/14	10
**Sample_G**	GW460	12/1/14	10

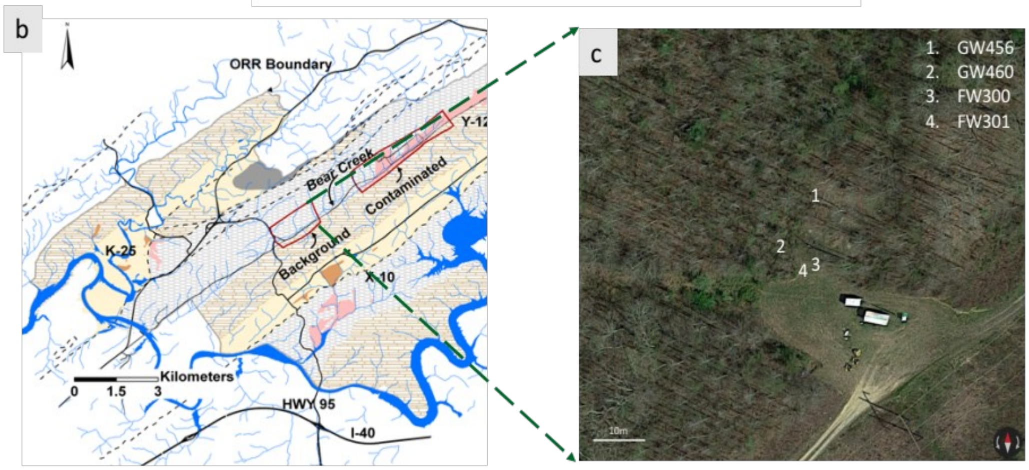

Supplement: Supplementary file 9 [file mBio.02899-18-sf003.docx]
